# Performance Evaluation of a Custom-Designed Contrast Media Injector in a 5-Tesla MRI Environment

**DOI:** 10.3390/bioengineering12060566

**Published:** 2025-05-25

**Authors:** Yuannan Hu, Wenbo Sun, Zhusha Wang, Wei Wang, Rufang Liao, Zhao Ruan, Huan Li, Haibo Xu, Daniel Topgaard

**Affiliations:** 1 Department of Radiology, Zhongnan Hospital of Wuhan University, Wuchang, Wuhan 430071, China; yuannanhu1991@163.com (Y.H.); sunwb3@mail2.sysu.edu.cn (W.S.); zushawang@whu.edu.cn (Z.W.); liaorufang@whu.edu.cn (R.L.); 2019183030036@whu.edu.cn (Z.R.); 2 Nanjing Jusha Display Technology Co., Ltd., Nanjing 210036, China; zongjingban@jusha.com.cn; 3 Department of Chemistry, Lund University, SE-221 00 Lund, Sweden; daniel.topgaard@fkem1.lu.se

**Keywords:** 5T, MRI, contrast media injector, RF interference, ultra-high field

## Abstract

The compatibility and safety of contrast media injectors (CMIs) at ultra-high magnetic field strengths remains a critical challenge. This study aimed to investigate a custom-designed CMI powered by a ceramic motor in a newly developed 5T MRI environment, comparing it with a commercial CMI commonly used in a clinic. Three key performance aspects of the CMI were assessed in the 5T environment: translational attraction force, injection flow rates, and total injected volume. Potential imaging artifacts were checked. The custom-designed CMI demonstrated robust performance in the 5T environment, maintaining injection accuracy across all test locations and ensuring translational attraction forces remained within safe thresholds, even in the most challenging positions. Importantly, the custom-designed CMI exhibited no significant radiofrequency (RF) interference, and no imaging artifacts were observed across routine clinical sequences. In contrast, the commercial 3T CMI showed RF interference in several sensitive tests, such as the gradient echo (GRE) sequence with a 0° flip angle and frequency-based detection methods, underscoring the need for field-specific CMI designs tailored to ultra-high field environments. Further tests were performed in monkey livers and a human brain in vivo. The custom-designed CMI proved to be safe, accurate, and fully compatible with the 5T environment.

## 1. Introduction

Cancers and vascular-related diseases, including acute ischemic stroke and myocardial infarction, remain among the leading causes of mortality worldwide [1]. Early diagnosis of these diseases is crucial in clinical practice, as timely intervention can significantly improve patient outcomes. Given the correlation between these diseases and vascular abnormalities, contrast-enhanced (CE) imaging where contrast media (CM) is injected into the vascular system plays a vital role in assessing blood supply to organs [2,3]. Since CM is an exogenous agent that circulates rapidly through the bloodstream, precise control over its dosage and injection rate is essential to ensure both safety and efficacy during CE imaging.

CM can be administered manually or via an automated contrast media injector (CMI). Automated CMIs are increasingly preferred in clinical practice due to their superior precision in controlling flow rates and timing, leading to more consistent imaging quality compared to manual injection [4]. Additionally, CMIs enable remote operation, avoiding exposure to X-rays, β-rays, or the acoustic noise generated by MRI scanners [5]. CMIs are widely utilized across various imaging modalities, including digital subtraction angiography (DSA), computed tomography (CT), positron emission tomography (PET), and magnetic resonance imaging (MRI). Among the above imaging modalities, MRI is a unique, non-invasive imaging tool offering exceptional soft tissue contrast. CE imaging further enhances MRI’s diagnostic utility, enabling more accurate tumor differentiation, classification, and grading [6]. It also provides valuable functional insights into semi-quantitative and quantitative kinetic parameters, improves visualization of vascular anatomy and ischemic lesions, and supports the evaluation of cancer treatment efficacy [7,8,9]. However, the MRI environment is a special room for electronic devices, due to its low compatibility for mental and high sensitivity for electromagnetic interference (EMI).

Recent advancements in MRI technologies have focused on achieving higher spatial resolution by transitioning clinical fields to higher magnetic field strengths (≥4T), such as 4.7T, 5T, 7T, 9.4T, 10.5T, and 11.7T [10]. Currently, 7T [11,12] and 5T [13,14] systems represent the two common clinical ultra-high-field MRI platforms. Traditionally, MRI systems at 3T and below have raised minimal concerns regarding CMI compatibility and safety. However, the growing adoption of ultra-high field MRI is anticipated to significantly enhance disease diagnosis and advance scientific research. Specifically, ultra-high magnetic fields introduce technical challenges, such as more pronounced EMI [15], which can severely degrade image quality. This issue becomes more serious when using CMIs near the scanner. Therefore, with the increasing prevalence of ultra-high field systems, rigorous testing is necessary to ensure that CMIs can function safely and effectively in these environments. To date, only limited evidence have been proven that clinical CMIs are compatible with ultra-high magnetic fields.

In the last few years, the novel 5T MRI scanner (uMR Jupiter, United-Imaging Healthcare, Shanghai, China) has garnered increasing attention due to its ability to perform whole-body scanning. In contrast, the 7T scanner is typically limited to head and extremity imaging due to safety concerns associated with high specific absorption rates (SARs) [16]. Additionally, the high cost of 7T systems restricts their accessibility. The 5T scanner strikes a balance between the 3T and 7T systems, offering the ability to perform whole-body imaging while providing significantly better image quality than 3T, not only for the head [13] but also for organs such as the pancreas [14], heart [17], kidney [18], and liver [19]. As the adoption of 5T scanners in clinical settings is expected to increase, concerns have emerged regarding the safety of using contrast media injectors (CMIs) at this field strength.

This study focused on evaluating the compatibility of a CMI on a recently developed 5T MR system by examining the following three critical aspects: (1) Safety: measuring translational attraction forces exerted by the static magnetic field when the CMI is positioned near the scanner; (2) Performance: assessing whether the high field affects the injection flow rate and total injected volume; (3) Image Quality: evaluating the potential impact of RF interference caused by the CMI on imaging performance. Additionally, we compared a custom-designed CMI with a commercial 3T CMI to find out whether the development of field-specific CMIs is necessary for ultra-high-field MRI systems.

## 2. Materials and Methods

**Figure 1:**
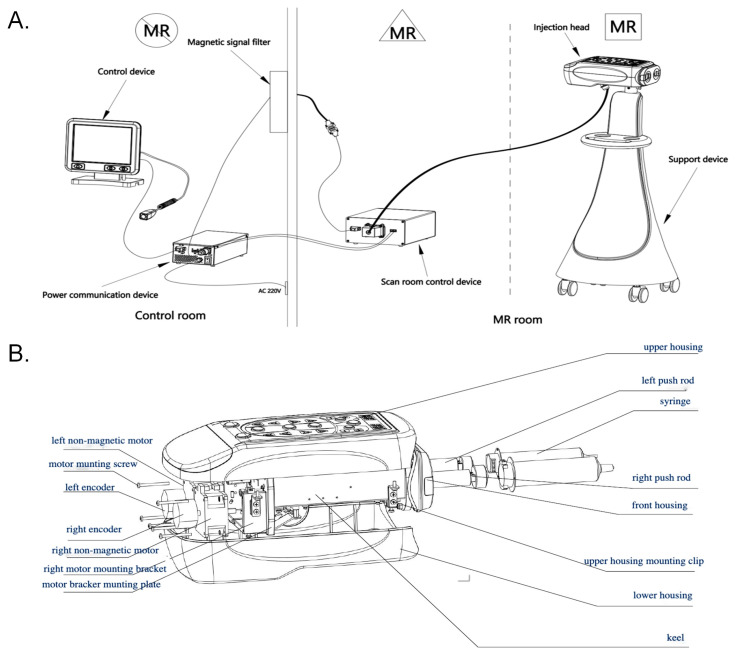
The diagram of the whole system of the designed CMI (**A**). It is driven by a ceramic motor located in the injection head (**B**).

All experiments were performed on a whole-body 5T MRI scanner in conjunction with a custom-designed CMI (Insight M12, Jusha Healthcare, Nanjing City, China). The CMI is powered by ceramic motors and consists of six main components: an injection head (where the ceramic motors are located), a support device, a control system for the scanning room, a power communication unit, a control display interface, and a filter, as shown in Figure 1A. The exploded view of the injection head is presented in Figure 1B. The injector head includes the upper and lower housings, the left and right ceramic motors and their encoders, the syringe-advancing mechanism, and the keel structure. The ceramic motors drive the push rods to inject and aspirate the contrast agent.

**Figure 2:**
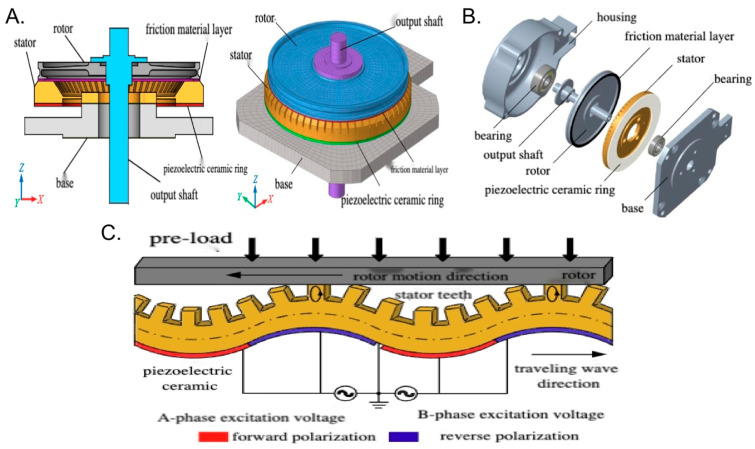
The side and vertical views as well as (**A**) the exploded view (**B**) of the ceramic motor and its simple working principle (**C**).

The ceramic motors are composed of several key parts: the housing, bearings, output shaft, rotor, friction material layer, stator (made from tin bronze), piezoelectric ceramic ring, and base, as shown in Figure 2A,B. The piezoelectric ceramic ring is bonded to the bottom of the stator, and the friction material layer is bonded to the rotor. The housing, bearings, and output shaft form the pre-load system, applying axial pre-load to the rotor and causing frictional contact between the rotor and stator teeth.

The motion process of the ceramic motor consists of two energy conversion stages. In the first stage, based on the inverse piezoelectric effect of the piezoelectric material, the input electrical energy is converted into high-frequency, small-amplitude vibrations of the stator elastomer. In the second stage, through the frictional interaction at the contact interface between the stator and rotor, the high-frequency, small-amplitude vibrations of the stator elastomer are converted into the rotational motion of the rotor. The working principle of the ceramic motor is mainly illustrated in Figure 2C. When two alternating voltages with the same amplitude, frequency, and a 90° phase difference in time are applied to the A and B regions of the piezoelectric ceramic ring, two standing wave modes with the same amplitude and a 90° phase difference in both time and space will be simultaneously excited. When these standing wave modes are superimposed, they form a traveling wave propagating along the circumferential direction of the stator. If appropriate pre-pressure is applied to the rotor, the elliptical motion of the particles on the stator’s tooth surface will drive the rotor to rotate, i.e., the small-amplitude vibrations of the stator are converted into the rotational motion of the rotor through frictional force.

The aim of this study was to evaluate three key aspects of CMI performance in the 5T MRI environment:

- Translational Attraction Force: The first objective was to measure and confirm that the translational attraction force exerted on the CMI by the static magnetic field remains below the established safety thresholds.
- Injection Accuracy: The second goal was to assess whether the RF pulses and high magnetic field would affect the CMI’s injection rate accuracy and the total injected volume. Injection flow rates and total injected volume were measured across multiple test conditions to determine if any deviations occurred.
- Image Artifact Evaluation: Finally, we tested whether the CMI would introduce any artifacts on the MR images during contrast agent injection. Standard clinical sequences were used to evaluate image quality, and the presence of any RF-induced artifacts was documented.

### 2.1. Measurement of the Translation Attraction Force

Prior to using the CMI in the MRI environment, it was essential to measure the translational attraction force acting on the device. For this, a tensile test was performed. A nylon rope was securely fastened around the CMI’s support device, with the other end connected to a calibrated tension meter. The tension meter was gradually pulled until the support device started to move, and the maximum recorded value was noted as the minimum force required to mobilize the CMI.

Next, the CMI was incrementally moved toward the MRI scanner, with the rope’s opposite end fixed to a stationary point, such as the scanner room door. The support device was slowly moved closer to the MRI bore, with tension measurements taken at regular intervals. The process continued until the CMI’s injection head was positioned near the bore of the 5T MRI system. The tensile value was recorded at each step. The translational attraction force was then calculated by subtracting the baseline force (measured far from the scanner) from the force recorded near the scanner.

### 2.2. Evaluation of the Accuracy of the Injection

The CMI was positioned at the worst-case location near the MRI scanner and equipped with two syringes: one for CM and one for saline. Both syringes were filled with distilled water at room temperature to simulate clinical conditions. The following parameters were evaluated:

(1) Accuracy of the injection rate

The injection rates were evaluated at three settings: minimum, 50% of the maximum, and maximum. For each rate, distilled water was injected, and the duration of injection was measured using a synchronized stopwatch. After each injection, the weight of the injected water was recorded using a precision balance and converted to volume. The deviation from the target injection rate was calculated by comparing the actual volume delivered with the expected volume. Each injection rate was tested three times to ensure consistency.

(2) Accuracy of the injection volume

The accuracy of the injection volume was tested at three dose settings: minimum dose (with the injection rate set to minimum), 50% of the maximum dose (with the injection rate at 50%), and maximum dose (with the injection rate at maximum). Similar to the injection rate test, the injected water was weighed and converted to volume, and the deviation from the target volume was calculated. Each volume setting was tested three times to ensure reliable measurements.

(3) Accuracy of the maximum injection pressure

The maximum injection pressure was tested by setting the pressure limit of the CMI to its maximum value (150 psi for injection head A and 100 psi for injection head B). A pressure gauge was connected to each injection head, and injections were performed at a rate of 2 mL/s. The pressure was recorded at the point when the device indicated that the pressure limit had been exceeded, causing the injection to stop. The deviation between the actual pressure and the set pressure limit was calculated to determine the accuracy of the pressure measurement. Each test was repeated three times for consistency.

### 2.3. Evaluation of the RF-Induced Image Artifacts

We investigated the potential RF interference caused by the custom-designed CMI (injector A) when used with the 5T MRI scanner. This was compared against a commercially available CMI (injector B), which is marketed as 3T-compatible. Both injectors were tested under four different conditions that simulate clinical use, which are as follows:

OFF: Injector closed and positioned away from the MRI scanner.

ON: Injector powered on but positioned away from the MRI scanner.

NEAR: Injector powered on and positioned near the MRI scanner.

**Figure 3:**
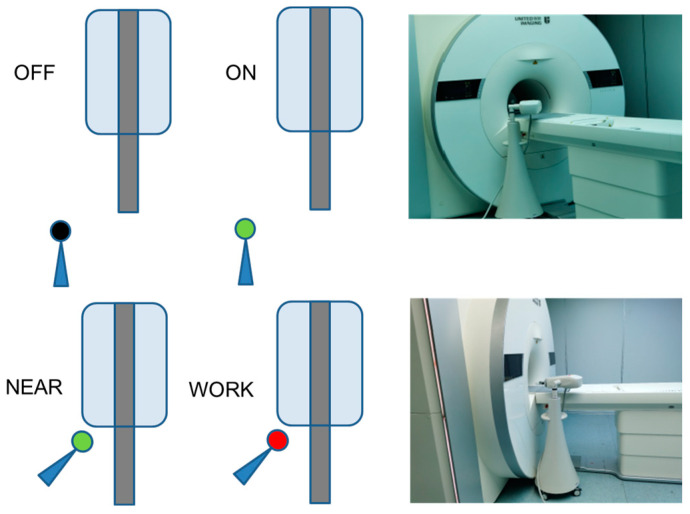
The diagram of four statuses of the injector. It has four statuses, OFF, ON, NEAR, and WORK.

WORK: Injector performing an injection at the highest speed (10 mL/s) and positioned near the scanner (at the bore, representing the worst-case scenario), as shown in Figure 3.

These conditions reflect the varying statuses of CMIs in a clinical environment. For ease of reference, these statuses are referred to as “OFF”, “ON”, “NEAR”, and “WORK” throughout the remainder of the text. To assess RF interference, the following tests were conducted:

- Test 1

Spin echo (SE) and gradient echo (GRE) sequences were scanned using a 48-channel head coil and a water phantom. Each sequence was scanned twice under all four conditions. The second scan was subtracted from the first to measure any changes in signal between the center and background areas. Two regions of interest (ROI) were drawn—one in the center and one in the background—to record the mean signal intensity and background noise across the four conditions.

- Test 2

Dynamic susceptibility contrast (DSC) and dynamic contrast-enhanced (DCE) imaging sequences were scanned with a 48-channel head coil and a water phantom. These sequences were scanned under all four statuses, and the signal in the background was measured and compared across the conditions. A single ROI was drawn in the background for signal intensity comparison.

- Test 3

A GRE sequence with a flip angle of 0° was scanned using a 24-channel body coil and a water phantom to assess potential RF interference. The mean signal in the background and any image artifacts were compared across the four statuses for both injectors. A single ROI was drawn in the background to quantify the signal.

- Test 4

A frequency-based detection method was employed to identify any RF interference and pinpoint its frequency. The amplitude and scale of the RF noise were compared to the signal threshold using only the VTC coil. The parameters for the SE, GRE, DSC, and DCE sequences used in these tests are detailed in Table 1.

- Test 5

With the custom-designed CMI, DCE scans were conducted in vivo at 5T on the livers of 20 monkeys, with changes in the background signal monitored to assess any variations associated with the injection. Additionally, a DSC scan was performed on two patients, and changes in the background signal were recorded. The MRI contrast agent Gadobutrol (Gadovist^®^, Schering AG, Berlin city, Germany) was injected during the scanning based on the weight of the animals and patients. This research has been approved and supported by the local ethics committee (Approved number 2022102).

### 2.4. Statistical Analysis

To assess the performance and reliability of the custom-designed CMI in the 5T MRI environment, a statistical analysis was conducted to compare the measurements obtained from the different tests. For the evaluation of injection accuracy, the measured values were compared with the expected values at both the nearest and most distant locations relative to the MRI scanner. For the phantom tests, we focused on comparing the mean signal intensities and standard variances of the ROIs in the four injector statuses: OFF, ON, NEAR, and WORK. For the in vivo scans, changes in the mean background signal intensities of the four corners of the images were recorded and compared between the periods with and without CM injection.

## 3. Results

### 3.1. Translation Attraction Force in the 5T MRI Environment

The CMI exhibited minimal magnetic attraction when positioned near the 5T MRI scanner. With the device in a closed state, the tensile force measured for the support device alone was 25 N. When the injection head was attached, the total tensile force increased to 27.5 N. Thus, the translational attraction force exerted on the injection head was calculated to be 2.5 N, which is well within the acceptable safety limits for MRI-compatible devices.

### 3.2. Accuracy of the Injector

#### 3.2.1. Accuracy of the Injection Rate and Volume

In the test of injection rate accuracy, the actual flow rate deviated minimally from the target rates across all conditions. As listed in Table 2, the differences between the target and actual flow rates remained within an acceptable range, ensuring reliable performance. For the injection volume, similar minimal deviations were observed, as outlined in Table 3. The actual injected volumes closely matched the target volumes, further confirming the accuracy of the CMI under different settings.

#### 3.2.2. Maximum Injection Pressure

When testing at an injection rate of 2 mL/s, the maximum injection pressure for the CM syringe across three trials was measured at 156.7 psi, 155.8 psi, and 156.3 psi, respectively. For the saline syringe, the measured maximum injection pressures were 103.6 psi, 105.5 psi, and 103.6 psi across three trials. These values fall within the acceptable range of the expected maximum pressure limits, indicating consistent and reliable performance for both syringes under 5T MRI conditions.

#### 3.2.3. RF Influence of the CMI on MRI Images

- Test 1

**Figure 4:**
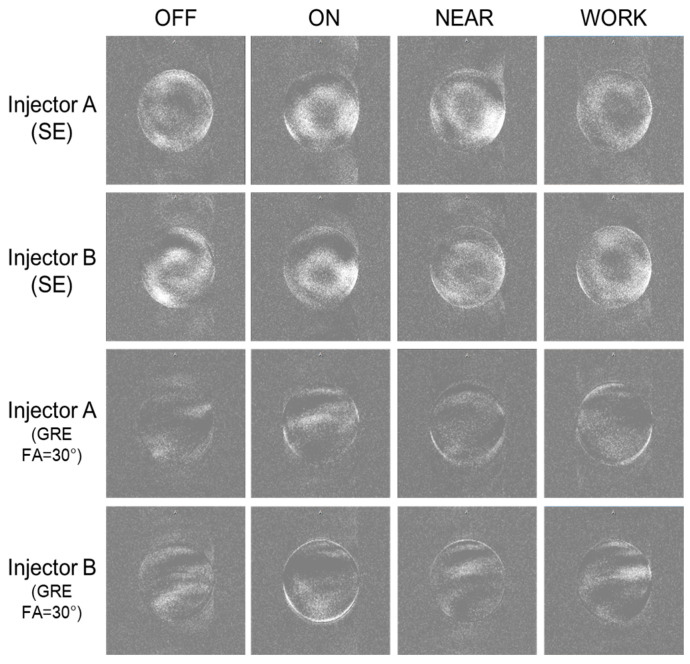
The subtracted images of the two injectors in four statuses for the test.

In this experiment, we compared the four statuses (OFF, ON, NEAR, WORK) for both SE and GRE sequences. For both sequences, there were no significant differences in the mean signal intensity of the subtracted images across the four conditions, as shown in Table 4 and Figure 4.

- Test 2

In DSC and DCE imaging sequences, notable differences were observed between the statuses “ON”, “NEAR”, and “WORK” when compared with the “OFF” status for both injectors. As shown in Table 5, Injector A exhibited a lower background signal compared to Injector B across all four conditions.

- Test 3

**Figure 5:**
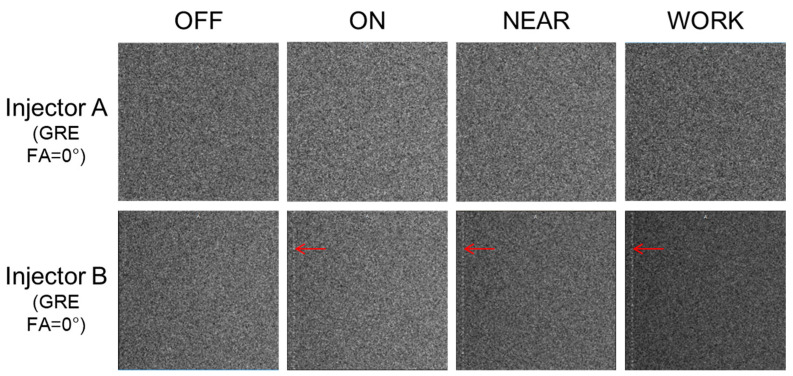
The scanned images of the two injectors in the four statuses for Test 3. The red arrow indicated RF interference of Injector B.

The GRE sequence with a flip angle of 0° was expected to be particularly sensitive to RF interference. Slight artifacts were observed only with Injector B, as shown in Table 5 and Figure 5. In all four statuses, Injector A demonstrated lower background signal intensity compared to Injector B.

- Test 4

**Figure 6:**
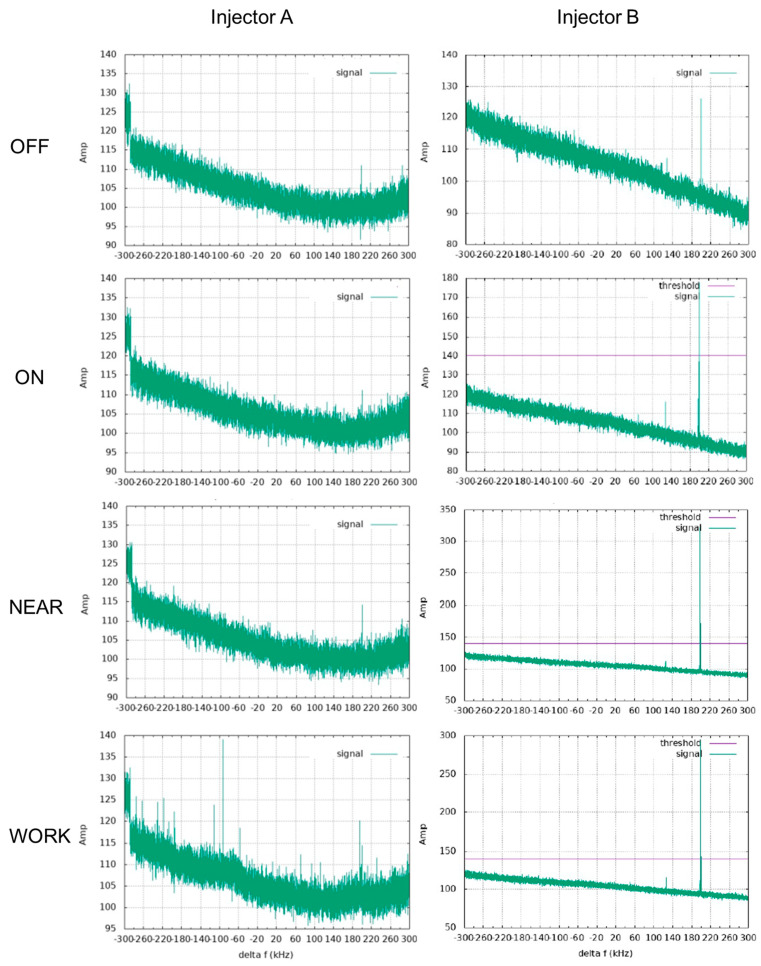
The RF interference test results of the two injectors in the four statuses for. The purple threshold means that the signal amplitude of the RF interference will not have any influence on the scanner, and here are 140 for all subfigures.

A frequency-based RF interference detection method was employed to further investigate the impact of the injectors in the MRI environment. This method, typically used for the VTC coil, can pinpoint the specific frequencies of RF interference. In the “ON”, “NEAR”, and “WORK” statuses, Injector B generated RF interference at frequencies around 130 kHz and 200 kHz, as shown in Figure 6. In contrast, no RF interference was detected for Injector A across all statuses.

- Test 5

**Figure 7:**
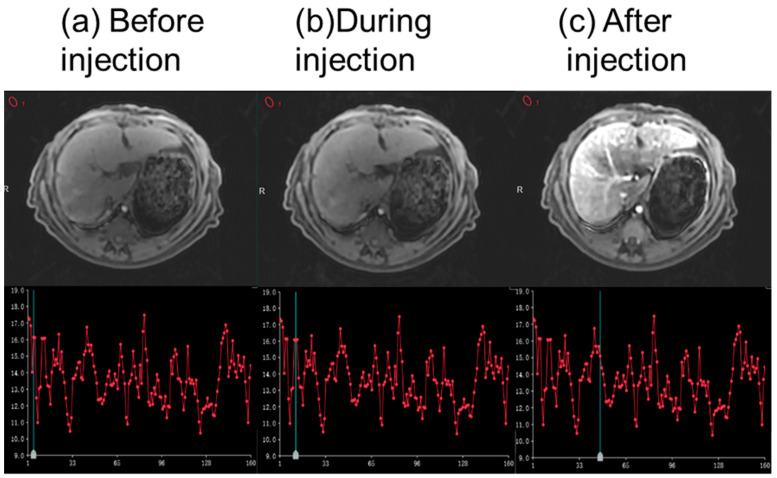
An example of the background signal intensity curve of the in vivo monkey liver DCE scans, showing no obvious changes during the injection. The green vertical lines indicate the phase of the images.

**Figure 8:**
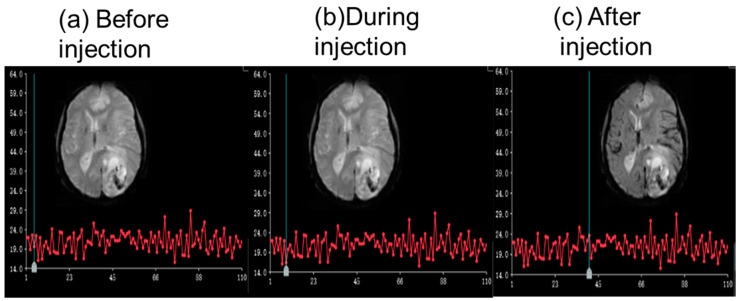
An example of the background signal intensity curve of a glioma patient (male, 59 years old), showing no obvious changes during the injection. The green vertical lines indicate the phase of the images.

There were no obvious changes in the background signal of the in vivo DCE scans, as shown in Figure 7. The mean ± SD signal of the background signal is 15.76 ± 2.45 before the injection, 14.42 ± 1.48 during the injection, and 15.02 ± 1.84 after the injection. No significant differences were found between different statuses (*p* > 0.05). For the in vivo DSC scans, similar findings were also confirmed, as shown in Figure 8.

## 4. Discussion

The uMR Jupiter is the first ultra-high field system cleared for whole-body applications by National Medical Products Administration (NMPA) and the U.S. Food and Drug Administration (FDA). The introduction of 5T ultra-high-field MRI scanner has raised concerns about the safety and compatibility of CMIs in these new high-field environments. In this study, we evaluated the performance of a custom-designed CMI and found that the 5T MRI scanner did not adversely impact the accuracy of the injection or cause significant translational attraction forces. Additionally, we compared the custom CMI with a commercially available CMI designed for 3T MRI through four different tests to assess RF interference. The results indicated that the commercial CMI generated RF interference, particularly in the GRE (flip angle = 0°) and frequency-based tests, while the custom CMI exhibited no significant interference.

MRI injection protocol is not as time-sensitive as for computed tomography (CT). However, compared with manual injection, injection by CMI is much more stable and repeatable with respect to the flow rate and timing of injection, and results in a significantly higher signal-to-noise ratio (SNR) and diagnostic image quality [5,20]. Moreover, research proved the feasibility of using a half-dose contrast agent at 5T MRI [14], making it possible to inject a volume of less than 3 mL CM in a patient weighing less than 60 kg, which could not be administered manually. In our study, even the minimum dose could be administered by CMI without obvious deviation from the actual dose, thus providing technical support for further research using a half-dose contrast agent.

Translational attraction poses safety risks in MRI environments [21]. We observed only a small translational attraction force of 2.5 N for the CMI at the worst-case location, indicating that the custom CMI is safe for use in the 5T environment. During injection tests, no significant differences were found between the two tested locations (a distant location and the worst-case scenario), indicating that the 5T scanner did not affect the CMI’s performance. This confirms that the custom CMI can reliably function in the 5T MRI environment. RF interference is a key factor that can degrade image quality in high-field MRI systems. In this study, SE and GRE sequences showed no significant RF interference from either CMI, as demonstrated by the absence of noticeable artifacts or signal degradation. Slight variations in background signals may have been caused by microflow within the phantom (Figure 4) rather than RF interference. Dynamic sequences, including DSC and DCE imaging, also revealed no noticeable artifacts with either CMI. These sequences are commonly used during contrast agent injections, and the findings are consistent with previous 7T MRI studies, which also demonstrated no artifacts during standard imaging procedures [22]. However, more sensitive methods, such as GRE with a 0° flip angle and frequency-based RF detection, revealed RF interference from the commercial 3T CMI, whereas the custom CMI remained free of interference. The GRE sequence with a small flip angle has been used to detect RF interference in hybrid PET/MRI systems [23]. Frequency-based tests further identified RF interference at approximately 130 kHz and 200 kHz from the commercial CMI, while the custom CMI exhibited no detectable interference under any condition.

These findings suggest that while standard imaging sequences (SE, GRE, DSC, DCE) may not detect RF interference at ultra-high fields due to the increased SNR and narrower bandwidth, more sensitive tests can uncover subtle artifacts. Such interferences, although minor, could affect sequences designed to detect subtle signal changes, such as blood oxygen level-dependent (BOLD) imaging [24]. Therefore, it is essential to use field-optimized CMIs, as demonstrated in this study and supported by the previous research on 7T MRI [22].

In this study, the in vivo DCE and DSC scans were also performed, and we found no difference in the background signal in each period (before injection, during injection, and after injection), these findings further confirmed the results of the previous tests. Since DCE and DSC scans are the two important sequences for the application of the CMI in the clinic [25,26], our findings therefore supported that a custom-designed CMI might be widely applied in an ultra-high field in the future.

A limitation of this study is that although we detected RF interference from the commercial 3T CMI, we were unable to isolate the specific component responsible for the interference. Additionally, we relied exclusively on imaging methods and did not employ electromagnetic interference (EMI)-sensing coils or other RF detection techniques [27]. Our tests were also conducted at a single center and involved only one commercial injector, which may limit the generalizability of our results. Future research should include multi-center clinical trials and more patients to validate these findings and further investigate the sources of RF interference.

Although 5T MRI systems are being integrated into clinical practice, traditional CMIs designed for 3T environments may still perform adequately in 5T systems with minimal RF interference. However, for more sensitive imaging sequences, such as BOLD, it remains critical to design and implement CMIs specifically optimized for 5T MRI systems to ensure the highest standards of performance and safety.

## 5. Conclusions

The custom-designed CMI demonstrated its capability to perform precise injection tasks while ensuring safety and maintaining full compatibility with the 5T MRI environment. These findings confirm the system’s reliability and suitability for seamless integration with ultra-high-field 5T MRI systems, providing reliable performance without compromising patient safety or image quality. This highlights its potential for clinical application in settings utilizing ultra-high-field 5T MRI systems, supporting optimal imaging outcomes during contrast-enhanced procedures.

## Figures and Tables

**Table 1:** Parameters of the MRI sequences.

Parameters	SE	GRE	DSC	DCE
TR (ms)	1500	800	1600	2.77
TE (ms)	30	25	32.5	1.03
FOV (mm^2^)	300 × 300	300 × 300	230 × 230	230 × 230
FA (°)	180	30/0	90	10
Gap (%)	0	0	20	0
Thickness (mm)	5	5	5	5
Slice number	1	1	19	24
Resolution (mm^2^)	1.17 × 1.17	1.56 × 1.56	1.80 × 1.80	1.60 × 1.60
BW (kHz)	1000	1000	1630	800
Accelerating factor	1	1	2	2
Number of Dynamic scans	1	1	50	50

TR = repetition time, TE = echo time, FOV = field of view, FA = flip angle; BW, bandwidth; SE = spin echo, GRE = gradient echo, DSC = dynamic susceptibility contrast, DCE = dynamic contrast-enhanced.

**Table 2:** Injection rate test.

Syringe	Target Rate (mL/s)	Target Volume (mL)	Injection Round	Time (s)	Volume (mL)	Actual Flow Rate (mL/s)	Average Actual Rate (mL/s)	Deviation of the Actual Rate (mL/s)
CM	0.1	60	1st	602.0	59.27	0.1	0.1	0
			2nd	602.6	59.32	0.1		
			3rd	604.3	59.45	0.1		
saline	0.1	60	1st	603.4	59.43	0.1	0.1	0
			2nd	603.2	59.25	0.1		
			3rd	603.7	59.60	0.1		
CM	5	50	1st	10.2	49.35	4.89	4.89	−0.11
			2nd	10.1	49.13	4.91		
			3rd	10.2	49.25	4.88		
saline	5	100	1st	20.2	99.38	4.94	4.95	−0.05
			2nd	20.1	99.21	4.96		
			3rd	20.2	99.36	4.94		
CM	10	50	1st	5.2	49.43	9.73	9.70	−0.3
			2nd	5.2	49.28	9.68		
			3rd	5.2	49.51	9.69		
saline	10	100	1st	10.2	99.36	9.84	9.84	−0.16
			2nd	10.2	99.41	9.84		
			3rd	10.2	99.25	9.83		

CM, contrast media.

**Table 3:** Injection volume test.

Syringe	Target Rate (mL/s)	Target Volume (mL)	Injection Round	Actual Volume (mL)	Average Actual Volume (mL)	Deviation of the Actual Volume (mL)
CM	0.1	1	1st	1.09	1.06	0.06
			2nd	1.04		
			3rd	1.05		
	5	30	1st	29.63	29.66	−0.34
			2nd	29.71		
			3rd	29.64		
	10	60	1st	59.38	59.39	−0.61
			2nd	59.35		
			3rd	59.44		
saline	0.1	1	1st	21.06	1.04	0.04
			2nd	1.03		
			3rd	1.04		
	5	55	1st	553	553	0.03
			2nd	551		
			3rd	556		
	10	110	1st	109.30	109.32	−0.68
			2nd	109.34		
			3rd	109.31		

CM, contrast media.

**Table 4:** The mean and SD of the signal at the center and background area for Test 1.

Sequence	Status	Injector A (Center)	Injector A (Background)	Injector B (Center)	Injector B (Background)
SE	OFF	15.27 ± 15.47	3.18 ± 4.68	12.85 ± 13.23	3.20 ± 4.70
	ON	13.88 ± 15.56	3.30 ± 4.80	15.32 ± 15.67	3.19 ± 4.79
	NEAR	12.51 ± 12.89	3.18 ± 4.65	16.7 ± 17.0	3.20 ± 4.70
	WORK	14.6 ± 14.4	3.0 ± 4.5	10.73 ± 12.02	3.33 ± 4.83
GRE (FA = 30°)	OFF	6.70 ± 8.45	2.54 ± 3.66	4.31 ± 6.72	2.32 ± 3.48
	ON	5.40 ± 8.19	2.40 ± 3.59	6.50 ± 8.67	2.46 ± 3.62
	NEAR	4.54 ± 6.99	2.38 ± 3.49	4.38 ± 6.57	2.28 ± 3.34
	WORK	5.77 ± 9.02	2.47 ± 3.59	5.22 ± 7.93	2.32 ± 3.54

SE = spin echo, GRE = gradient echo, OFF: Injector closed and positioned away from the MRI scanner, ON: Injector powered on but positioned away from the MRI scanner, NEAR: Injector powered on and positioned near the MRI scanner, WORK: Injector performing an injection at the highest speed (10 mL/s) and positioned near the scanner.

**Table 5:** The mean and SD of the signal at the whole image area for Test 2 and 3.

Sequence	Status	Injector A	Injector B
DCE	OFF	21.11 ± 0.67	23.61 ± 0.46
	ON	20.96 ± 0.58	23.49 ± 0.46
	NEAR	20.92 ± 0.63	23.46 ± 0.51
	WORK	21.01 ± 0.60	23.48 ± 0.45
DSC	OFF	41.02 ± 0.99	48.84 ± 1.28
	ON	41.23 ± 0.90	48.96 ± 1.15
	NEAR	40.86 ± 0.82	48.15 ± 1.23
	WORK	41.26 ± 0.95	48.32 ± 1.48
GRE (FA = 0°)	OFF	42.35 ± 4.12	55.45 ± 6.99
	ON	42.35 ± 4.14	56.00 ± 7.79
	NEAR	42.42 ± 4.14	56.4 ± 8.3
	WORK	42.55 ± 4.18	56.9 ± 9.6

OFF: Injector closed and positioned away from the MRI scanner, ON: Injector powered on but positioned away from the MRI scanner, NEAR: Injector powered on and positioned near the MRI scanner, WORK: Injector performing an injection at the highest speed (10 mL/s) and positioned near the scanner.

## Data Availability

The raw data supporting the conclusions of this article will be made available by the authors on request.

## Abbreviations

The following abbreviations are used in this manuscript:

BOLD	Blood oxygen level-dependent
CM	Contrast media
CMI	Contrast media injector
CE	Contrast-enhanced
MRI	Magnetic resonance imaging
RF	Radiofrequency
EMI	Electromagnetic interference
SE	Spin echo
GRE	Gradient echo
DSC	Dynamic susceptibility contrast
DCE	Dynamic contrast-enhanced
